# Role of SCC*mec* type in resistance to the synergistic activity of oxacillin and cefoxitin in MRSA

**DOI:** 10.1038/s41598-017-06329-2

**Published:** 2017-07-21

**Authors:** Nathalie T. Reichmann, Mariana G. Pinho

**Affiliations:** Instituto de Tecnologia Química e Biológica António Xavier, Universidade Nova de Lisboa, Oeiras, Portugal

## Abstract

β-lactam antibiotics target penicillin-binding proteins (PBPs) preventing peptidoglycan synthesis and this inhibition is circumvented in methicillin resistant *Staphylococcus aureus* (MRSA) strains through the expression of an additional PBP, named PBP2A. This enzyme is encoded by the *mecA* gene located within the Staphylococcal Chromosome Cassette *mec* (SCC*mec*) mobile genetic element, of which there are 12 types described to date. Previous investigations aimed at analysing the synergistic activity of two β-lactams, oxacillin and cefoxitin, found that SCC*mec* type IV community-acquired MRSA strains exhibited increased susceptibility to oxacillin in the presence of cefoxitin, while hospital-acquired MRSA strains were unaffected. However, it is not clear if these differences in β-lactam resistance are indeed a consequence of the presence of the different SCC*mec* types. To address this question, we have exchanged the SCC*mec* type I in COL (HA-MRSA) for the SCC*mec* type IV from MW2 (CA-MRSA). This exchange did not decrease the resistance of COL against oxacillin and cefoxitin, as observed in MW2, indicating that genetic features residing outside of the SCC*mec* element are likely to be responsible for the discrepancy in oxacillin and cefoxitin synergy against these MRSA strains.

## Introduction

The human pathogen *Staphylococcus aureus* is a leading cause of infections ranging from superficial wound infections to more serious illnesses including bacteremia, endocarditis or toxic shock syndrome^1^. Patient treatment commonly involves the use of β-lactams, antibiotics that prevent cell wall synthesis by targetting the four *S. aureus* penicillin-binding proteins (PBPs) responsible for the transpeptidation of the peptidoglycan^2^. The use of methicillin, an early semisynthetic β-lactam, was soon followed by the emergence of methicillin resistant *S. aureus* (MRSA). Today, MRSA strains demonstrate resistance to multiple antibiotics and include not only hospital-acquired (HA-MRSA), but also the later emerging community-acquired (CA-MRSA) strains, which tend to be more virulent^3^.

The key determinant of β-lactam resistance is the expression of PBP2A, an additional PBP that has low affinity for β-lactams, thereby maintaining transpeptidation activity in the presence of otherwise lethal concentrations of these antibiotics^4^. PBP2A is encoded by *mecA*, a gene located within a mobile genetic element called the Staphylococcal Chromosome Cassette *mec* (SCC*mec*)^5^. To date, there are 12 SCC*mec* types described, varying greatly in size (~21 kb to 67 kb) and most commonly HA-MRSA strains carry SCC*mec* types I, II and III, while CA-MRSA strains have SCC*mec* types IV and V^6–8^. All MRSA strains possess a *mec* gene complex, a cassette chromosome recombinase (*ccr*) gene complex and junkyard or joining (J) regions^8^. The *mec* gene complex includes *mecA* and its regulatory genes *mecI* and *mecR1*, though depending on the SCC*mec* type, these regulatory genes may be disrupted by insertional inactivation sequences^9^. The *ccr* gene complex encodes site-specific recombinases responsible for the integration and excision of the SCC*mec* at the 3′ end of the *orfX* gene, referred to as the *attB* site^10^. While this site is well-defined, the mechanism of integration and excision and the acquisition of the genetic element itself are still not fully elucidated and many of its ORFs have not been well characterised. Homology to genetic regions identified in *Staphylococcus sciuri*, *Staphylococcus fleuretti*, *Staphylococcus xylosus*, *Staphylococcus hominis*, and *Macrococcus caseolyticus* makes them all possible SCC*mec* sources, and although the exact mechanism of SCC*mec* acquisition remains unknown, one possibility is via bacteriophage-mediated transduction^11–15^. *S. aureus* strains carry a huge array of bacteriophages, which are thought to play a key role in the transfer of DNA within the species^16^. In fact, it has been shown that the *mec* genes can be introduced into MSSA backgrounds by transduction^17^. However, no bacteriophages have been shown to transfer DNA between different staphylococcal species, supporting the idea of SCC*mec* acquisition via conjugation^18^.

The increase in MRSA incidence has led to a need for alternative therapies, the focus of which has been not only the identification of new antibiotics with novel killing mechanisms, but also the study of synergistic activity of currently available drugs. One such example is the use of two β–lactams, oxacillin and cefoxitin, which have highest affinity for different PBPs (PBP1/PBP2, and PBP4, respectively)^19^. Addition of cefoxitin reduces the minimum inhibitory concentration (MIC) of oxacillin in CA-MRSA SCC*mec* type IV strains MW2 and USA300, suggesting that PBP4 is required for β–lactam resistance in these strains^19^. Accordingly, genetic inactivation of *pbpD* encoding PBP4 was also found to decrease resistance to oxacillin^19^. Surprisingly, this effect is not observed in HA-MRSA SCC*mec* type I strain COL, and further blind testing of clinical isolates found that all tested type IV SCC*mec* strains demonstrated a synergistic oxacillin and cefoxitin inhibitory effect, while HA-MRSA strains did not^19, 20^. It was therefore posited that the differences in β-lactam resistance observed in CA-MRSA and HA-MRSA strains may be due to differences in the genetic composition of the SCC*mec* type.

In this work, we aimed to analyse the effects of exchanging the SCC*mec* type I of COL with type IV of MW2, in order to determine whether the resistance of HA-MRSA strain COL to the synergistic action of oxacillin and cefoxitin was specifically dependent on the type of SCC*mec*. The results shown here indicate that the genetic differences between SCC*mec* type I and type IV do not significantly alter the resistance level or the morphological response of COL to the challenge of these β-lactams, indicating that there are additional key genetic factors involved.

## Materials and Methods

### Bacterial strains and growth conditions

All strains used in this study are listed in Table 1. *E. coli* strains were grown at 37 °C in Luria-Bertani broth (LB, Difco) or on LB agar (LA, Difco) supplemented with 100 μg/ml of ampicillin (Apollo Scientific), where appropriate. *S. aureus* strains were grown at 37 °C or 30 °C where indicated, in tryptic soy broth (TSB, Difco) or on tryptic soy agar (TSA, Difco), with the addition of 10 μg/ml of erythromycin (Apollo Scientific) or 10 μg/ml of tetracycline (Sigma-Aldrich) when necessary.

**Table 1:** Bacterial strains used in this study.

Strain	Relevant Features	Reference
**Plasmids**		
pSR	Multi-copy plasmid encoding *ccrA2* and *ccrB2* genes of N315; TetR	10
pMAD	*E. coli*/*S. aureus* allelic exchange vector; AmpR, EryR	23
pMAD-MW2Δ*spa*	600b of up- and downstream regions of the *spa* gene of MW2 cloned into pMAD; AmpR, EryR	This study
pMAD-SACOL0057	SACOL0057 inserted between 650 bp of the up- and downstream regions of *attB* site of MW2 cloned into pMAD; AmpR, EryR	This study
***Escherichia coli***		
DC10B	Δ*dcm* in DH10B background; Dam methylation only; for cloning	51
***Staphylococcus aureus***		
RN4220	restriction-negative derivative of 8325-4	52
COL	HA-MRSA; TetS; SCC*mec* type I, ST250, pbla^−^	28
COL-S	COL with SCC*mec* type I excised, pbla^-^	28
MW2	CA-MRSA; SCC*mec* type IV, ST1, pbla^+^	53
COL type IV	COL with SCC*mec* type IV, pbla^-^	This study
RN4220 pPBP4-J	pPBP4-J integrated into RN4220, insertionally inactivating *pbpD* encoding PBP4; EryR	22
COL *pbpD*mut	pPBP4-J integrated into COL, insertionally inactivating *pbpD* encoding PBP4; EryR; pbla^-^	This study
MW2 *pbpD*mut	pPBP4-J integrated into MW2, insertionally inactivating *pbpD* encoding PBP4; EryR; pbla^+^	This study
COL type IV *pbpD*mut	pPBP4-J integrated into COL type IV, insertionally inactivating *pbpD* encoding PBP4; EryR; pbla^-^	This study
MW2-S	MW2 with SCC*mec* type IV excised; pbla^+^	This study
MW2-SΔ*spa*	MW2 with SCC*mec* type IV excised and deletion of *spa* gene; pbla^+^	This study
MW2-SΔ*spa*0057	MW2 with SCC*mec* type IV excised, deletion of *spa* gene and insertion of *SACOL0057* downstream of *orfX*; pbla^+^	This study

### Strain construction

For construction of COL type IV, MW2 DNA was transferred to COL-S (lacking SCC*mec*) by transduction. Bacteriophage 80α was used to infect the donor strain, MW2, and the lysate was collected and sterilised by filtration. The lysate was next incubated with COL-S, the recipient strain, agitated for 20 min at 37 °C, and COL type IV was selected for on a plate containing 12 µg/ml oxacillin in the bottom 10 ml 0.3GL agar, followed by 20 ml 0.3GL agar without antibiotics (giving an average oxacillin concentration of 4 µg/ml)^21^. COL type IV was confirmed by next generation sequencing (NGS).

For excision of the SCC*mec* element of MW2, plasmid pSR was transduced from RN4220 into MW2 using bacteriophage 80α and selecting for tetracycline resistance, as previously described^10^. This strain was then grown in TSB and back-diluted 1:500 every 12 hrs for a total of 70 hrs, before plating on TSA. Colonies were patched on TSA as well as TSA supplemented with 4 µg/ml oxacillin to confirm the loss of SCC*mec*, and 10 µg/ml tetracycline to confirm the loss of plasmid pSR, before PCR confirmation (primers 11/12 for pSR; primers 13/8 for SCC*mec* excision) and sequencing of the SCC*mec* excision region.

For construction of PBP4 mutants, plasmid pPBP4-J^22^ was transduced from RN4220 into COL, MW2 and COL type IV using bacteriophage 80α and erythromycin selection, as previously described^21^, resulting in COL *pbpD*mut, MW2 *pbpD*mut and COL type IV *pbpD*mut, respectively.

Primers used in this study are listed in Supplementary Table S1. For construction of MW2-SΔ*spa*, the 600 bp upstream and downstream of the *spa* gene were amplified from MW2 chromosomal DNA using primer pairs 1/2 and 3/4, respectively. The PCR fragments were then fused by overlap PCR using primers 1/4, resulting in a single 1.2 kb PCR fragment. Following digestion with *Eco*RI/*Bam*HI, this PCR fragment was ligated to pMAD, which had been similarly digested. This plasmid, pMAD-MW2Δ*spa*, was initially obtained in DC10B, confirmed by sequencing and electroporated into RN4220 at 30 °C (the permissive temperature), selecting for erythromycin resistance, as previously described^21, 23^. Bacteriophage 80α was used to transduce this plasmid into MW2-S, and integration and excision using erythromycin selection was performed as previously described^21^. Deletion of the *spa* gene was confirmed by sequencing and this strain was named MW2-SΔ*spa*.

For construction of MW2-SΔ*spa*0057, the 650 bp upstream and downstream of the *attB* site were amplified from MW2 chromosomal DNA using primer pairs 5/6 and 9/10, respectively. The 550 bp downstream of the *attB* site of COL (encoding SACOL0057) were amplified from COL chromosomal DNA using primer pair 7/8. The three PCR fragments were then fused by overlap PCR using primer pair 5/10. Both pMAD and the PCR product were digested with *Bgl*II/*Bam*HI and ligated, before recovery in DC10B and confirmation by sequencing. The plasmid, named pMAD-SACOL0057, was next introduced into RN4220 at the permissive temperature (by erythromycin selection), transduced using bacteriophage 80α into MW2-SΔ*spa* and following integration and excision, strain MW2-SΔ*spa*0057 was obtained.

### Next Generation Sequencing (NGS)

Chromosomal DNA was purified from COL and COL type IV using a standard phenol-chloroform extraction technique^24^. DNA was sequenced using the Illumina MiSeq system at Instituto Gulbenkian de Ciência, Oeiras, Portugal, producing 300 bp paired end reads with over 100x average coverage. The sequence reads of COL were assembled with SeqMan NGen 12 software (DNASTAR, Inc) using the COL genome (NCBI Accession NC_002951.2) as a reference. This sequence was then used as a reference for assembly of COL type IV, where single nucleotide polymorphisms (SNPs) with a consensus below 50% were excluded from SNP analysis, those with 50–85% consensus were confirmed by region specific sequencing by GATC Biotech and those above 85% were accepted.

### Minimum Inhibitory Concentration (MIC) determination by Population Analysis Profiles (PAPs) and microdilution

PAPs were performed to assess the MIC of strains against oxacillin, cefoxitin and a combination of both. Overnight cultures of *S. aureus* strains were diluted tenfold from 10^−1^ to 10^−7^ and 10 µl of each dilution as well as the non-diluted culture was spread on a TSA plate containing twofold dilutions of 0.0625–1,024 µg/ml oxacillin (Sigma), or 1–1,024 µg/ml cefoxitin (Sigma). Plates were incubated at 37 °C for 48 hrs and colony forming units (CFU) were counted. The MIC was defined as the concentration at which growth of 99.9% of the population was inhibited, therefore causing a 3 log drop in CFU/ml. For testing of synergistic activity, twofold dilutions of oxacillin were tested in the presence of ¼ X MIC of cefoxitin (8 or 64 µg/ml), similar to previous experiments as described by Memmi *et al*. (2008).

MIC determination by microdilution was performed in 96-well plates, where overnight cultures were diluted to a final OD_600 nm_ of 0.0025 (cell density of ~ 5×10^5^ CFU/ml) in wells containing twofold dilutions of the following antibiotics in TSB: 2–1,024 µg/ml cephradine (Sigma), 0.25–64 µg/ml vancomycin (Sigma), 2–1,024 µg/ml bacitracin (Sigma), 2–1,024 µg/ml D-cycloserine (Apollo Scientific), 0.25–64 µg/ml daptomycin (Cubist Pharmaceuticals) in the presence of 50 μg/ml of Ca^++^, 0.25–64 µg/ml chloramphenicol (Sigma), 2–1,024 µg/ml nalidixic acid (Sigma). Plates were incubated at 37 °C and assessed for growth after 48 hrs, whereby the MIC was defined as the lowest concentration of antibiotic at which growth was prevented.

### Total protein extraction and western blot analysis

Extraction of proteins and western blotting were performed as previously described, with minor alterations^25^. In brief, *S. aureus* strains were grown in 50 ml TSB with and without 0.5 µg/ml oxacillin (and with 10 µg/ml erythromycin for *pbpD*mut strains) to an OD_600 nm_ of 0.8. Cells were lysed using glass beads in a Fast Prep FP120 (Thermo Electro Corporation), and separated from the glass beads by centrifugation for 1 min at 4,200 rpm, before removal of unbroken cells by centrifugation for 15 min at 16,000 × g. Protein content was quantified using the BCA protein assay kit (Pierce) and 20 mg of protein extract were loaded in each well of a 10% SDS-PAGE gel and separated by 80 V for 4 hrs. Samples were transferred to a Hybond-P Polyvinylidene difluoride (PVDF) membrane (GE Healthcare) using a semidry transfer cell (Biorad), and cut along the 50 kDa marker to separate the PBP2A and MreC containing regions. Each membrane was blocked for 1 hr with 5% milk in PBST (0.5% Tween 20 in phosphate buffered saline) before incubation at 4 °C with anti-PBP2A (Slidex MRSA detection, Biomerieux) or anti-mreC antibody^26^ overnight. Washed membranes were then incubated for 1 h with HRP-conjugated goat anti-mouse and HRP-conjugated goat anti-rabbit secondary antibodies (GE Healthcare), respectively. Bands were visualised using the ECL Plus Western blotting detection kit (Amersham) and Chemidoc XRS + Imaging System (Biorad).

### Structured Illumination Microscopy (SIM)

Overnight cultures of COL and COL type IV were back-diluted 1:200 in 20 ml TSB and grown to an OD_600 nm_ of 0.2, before 5 ml aliquots were transferred to test tubes. Cultures were grown for a further 1 hr, agitated at 37 °C, in the presence or absence of oxacillin (256 µg/ml for COL; 512 µg/ml for COL type IV) and 1 ml of culture was then pelleted, washed in 1 ml PBS and incubated at 37 °C agitated for 5 min with 1 µg/ml Hoechst 33342 (Invitrogen), 10 µg/ml Nile Red (Invitrogen) and 0.8 µg/ml BODIPY FL conjugated vancomycin (Van-FL, Molecular Probes). Cells were then pelleted and washed with PBS before being mounted on a 1.2% PBS agarose pad and SIM imaging was performed, due to its improved resolution compared to conventional microscopy, using a Plan-Apochromat 63x/1.4 oil DIC M27 objective, in an Elyra PS.1 microscope (Zeiss) with a Pco.edge 5.5 camera. Images were acquired using five grid rotations, with grating periods of 34 µm period for 561 nm laser (100 mW), 28 µm period for 488 nm laser (100 mW) and 23 µm period for 405 nm laser (50 mW) and images were reconstructed using ZEN software (black edition, 2012, version 8.1.0.484) based on a structured illumination algorithm, using synthetic, channel specific optical transfer functions, as described previously^27^.

### Data availability

NGS of COL type IV identified SNPs indicated in Table S2.

## Results

### Exchange of SCC*mec* Type I for Type IV in HA-MRSA strain COL

**Figure 1:**
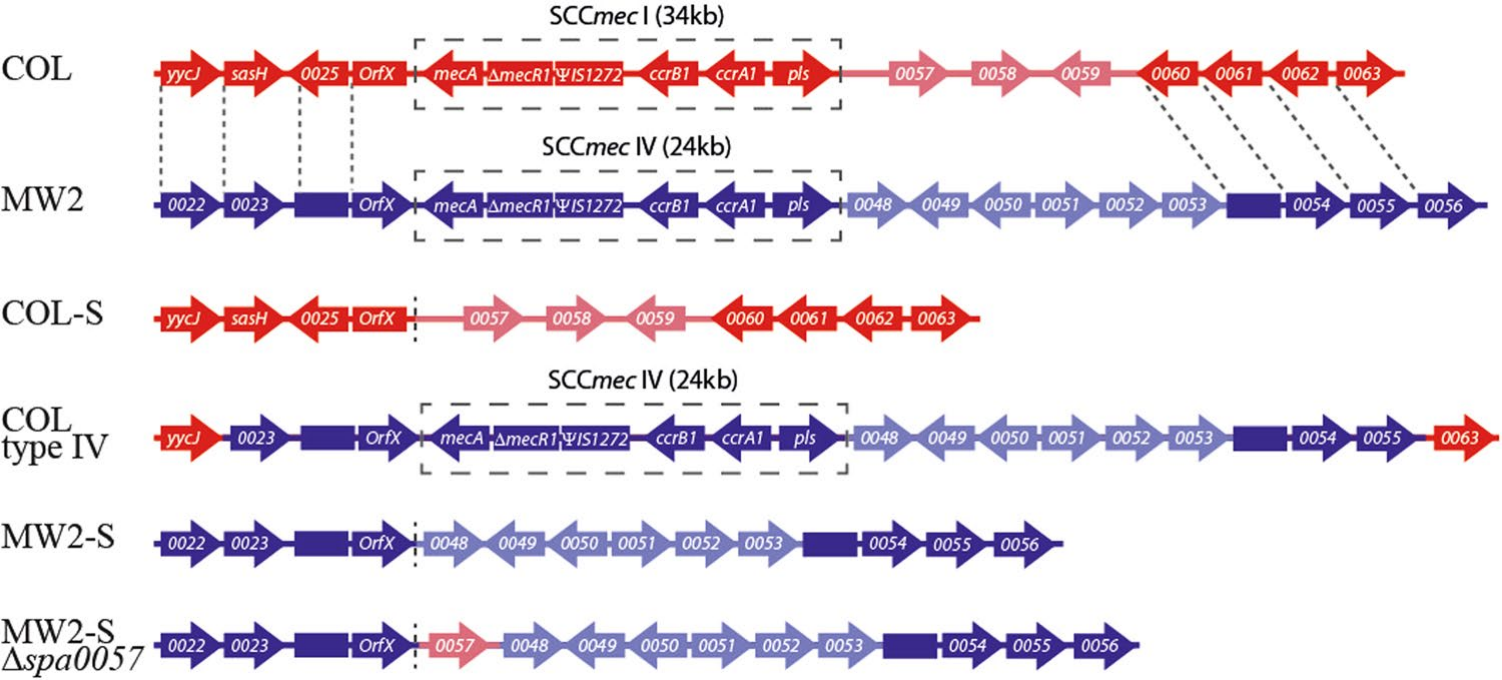
Schematic representation of *S. aureus* strains with SCC*mec* types I and IV. The SCC*mec* element and surrounding regions of strains COL, MW2, COL type IV, COL-S, MW2-S and MW2-SΔ*spa*0057 are graphically represented here. Annotated ORFs are shown as arrows and non-annotated homologous regions are represented as boxes. The SACOL ORFs are colored in red, the MW ORFs are in blue and the SCC*mec* element is highlighted by a black box (not all SCC*mec* genes are shown). Homologous regions of COL and MW2 genomes are shown in dark colors and indicated by dotted lines, while non-homologous regions are in light colors. Next generation sequencing of COL type IV indicates a double crossover event occurring downstream of *yycJ* (*mw0022* homologue) and upstream of *sacol0063* (*mw0056* homologue). Not to scale.

In order to investigate the specific differential roles of SCC*mec* types I and IV in susceptibility to the action of combined oxacillin and cefoxitin in *S. aureus*, it was necessary to analyse these mobile genetic elements in the same genetic background. Therefore we decided to attempt the exchange of the native SCC*mec* type I in COL (MIC of 512 µg/ml) for the SCC*mec* type IV from MW2. The SCC*mec* of COL had previously been excised^28^ and the COL-S strain (MIC of 2 µg/ml) served as the recipient strain while MW2 was the donor strain for transductions using bacteriophage 80α. Since the acquisition of type IV SCC*mec* could confer a low level of oxacillin resistance, selection was performed with 4 µg/ml oxacillin. We were able to obtain a COL type IV clone, which was identified by PCR and confirmed by Next Generation Sequencing (NGS). NGS showed that in addition to the exchange in SCC*mec* type, the immediate downstream region in strain COL type IV carried non-homologous genes *mw0048*-0*053* and lacked *sacol0057-0059* (Fig. 1). Compared to the sequenced COL genome, there were an extra 96 SNPs, all of which resided within 5 kb of the SCC*mec* element and were identical to the MW2 genome (see Supplementary Table S2). This suggests that the bacteriophage 80α took up 34 kb of MW2 chromosomal DNA, which encoded the SCC*mec* genetic element (24 kb) and approximately 10 kb of flanking regions, and that the insertion of the SCC*mec* sequence into COL-S may have taken place via a double crossover event (Figure 1).

One additional genetic modification in the genome of COL type IV compared to COL was found to be a deletion within the *map* gene, encoding the Major Histocompatibility Complex Class II Analog protein (Map), also referred to as the extracellular adherence protein (Eap)^29, 30^. This protein is a member of the secretable expanded repertoire adhesive molecules (SERAM) family, which has been linked to roles in adherence to fibroblasts, activation of proinflammatory cytokines, and impaired wound healing *in vivo* ^31–33^. The protein shows size variability across *S. aureus* clinical strains, with four to six tandem repeats, and the mutation in COL type IV resulted in the deletion of one out of five tandem repeats. The *map* gene of COL-S was sequenced and found to also contain this deletion, indicating that this modification did not arise as a result of the acquisition of SCC*mec* type IV, but was already present in the recipient strain. Therefore, given that Map has not previously been associated with β-lactam resistance or SCC*mec* acquisition, and its natural size variation, it is highly unlikely that this deletion will affect the findings put forward in this paper.

**Figure 2:**
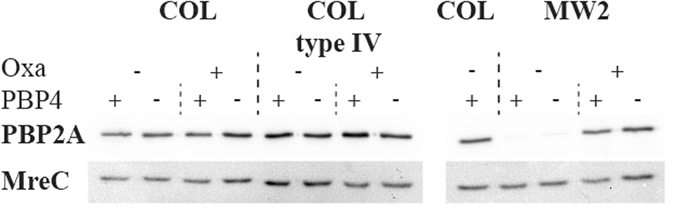
Western blot analysis of PBP2A expression levels. Total protein extracts of COL, COL type IV, MW2 and their PBP4 mutants grown in the presence and absence of 0.5 µg/ml oxacillin (Oxa) were separated by 10% SDS-PAGE gel and assessed for PBP2A expression using anti-PBP2A antibody (top panel). The same PVDF membrane was probed using anti-mreC antibody as a loading control (bottom panel).

Having constructed COL type IV, we wanted to assess the basal level of expression of PBP2A. Both type I and type IV SCC*mec* contain insertional inactivations within the *mec* inducer and repressor (*mecIR*) and in strain COL, this results in constitutive expression of *mecA* ^9, 19^. However, in MW2, the presence of the β-lactamase carrying plasmid, p*bla*, causes repression of *mecA* transcription in the absence of β-lactams, and the loss of this plasmid leads to constitutive *mecA* expression^19, 34^. Since COL type IV does not carry p*bla* (as confirmed by NGS), it was expected to have high basal levels of PBP2A expression. Western blot analysis confirmed that PBP2A levels were similar in COL type IV compared to COL and did not significantly increase following oxacillin challenge (Fig. 2). In contrast, detection of PBP2A in MW2 required the addition of oxacillin (Fig. 2), as previously shown^19^. These results confirmed that the type IV SCC*mec* does not carry *mecA* regulatory elements capable of repressing PBP2A expression.

### Unaltered resistance of COL type IV versus COL to the synergistic effect of oxacillin and cefoxitin

**Figure 3:**
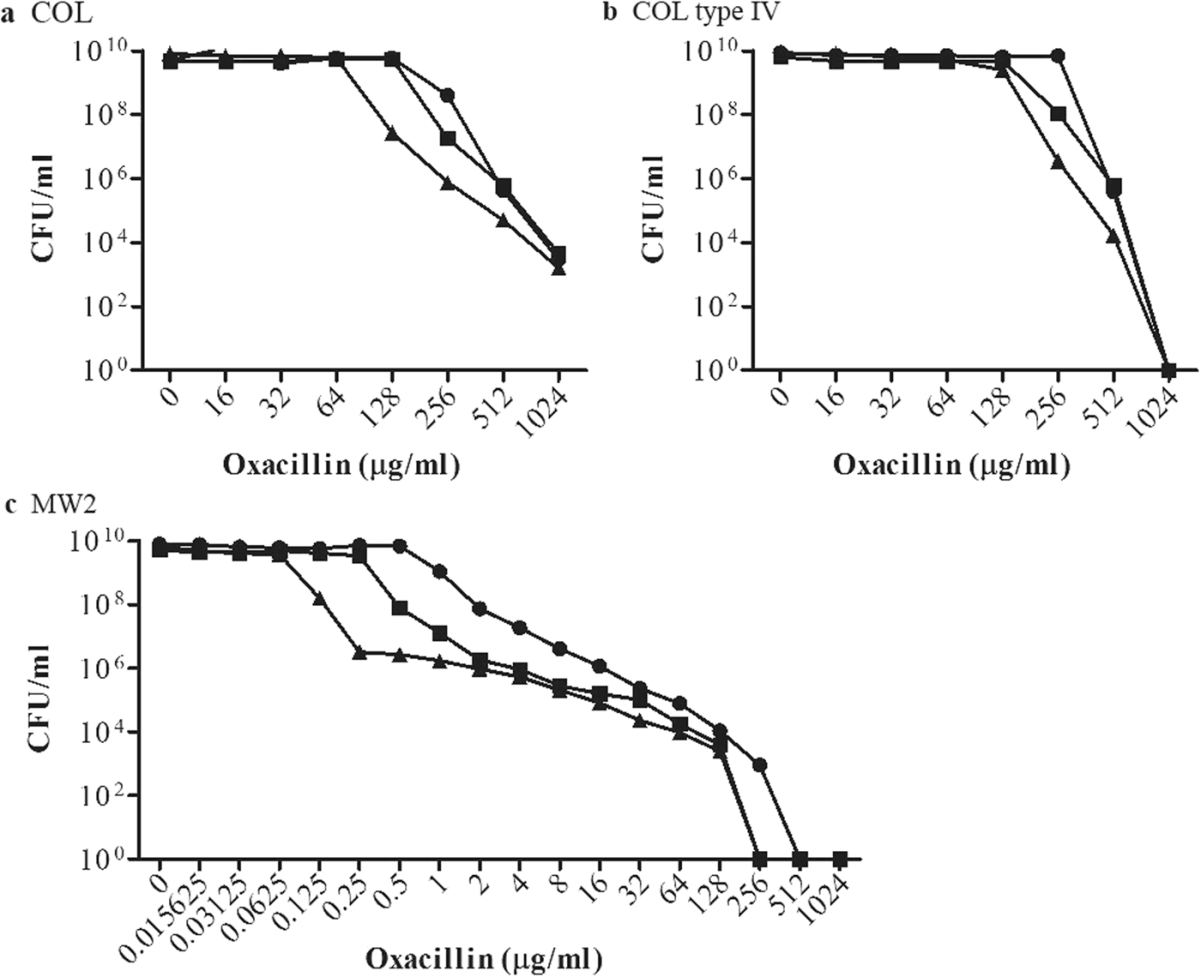
Population Analysis Profiles of *S. aureus* susceptibility to oxacillin in the presence or absence of cefoxitin. Oxacillin susceptibility was tested in strains (**a**) COL, (**b**) COL type IV, and (**c**) MW2, shown as circles. Corresponding strains lacking PBP4 are represented as squares and strains grown in the presence of ¼ X MIC of cefoxitin are shown as triangles (64 µg/ml for COL and COL type IV; 8 µg/ml for MW2). Strains were plated on agar containing twofold dilutions of oxacillin, incubated at 37 °C for 48 h and the number of colony forming units (CFU) per ml was calculated. The experiment was performed in triplicate and a representative graph is shown here.

**Figure 4:**
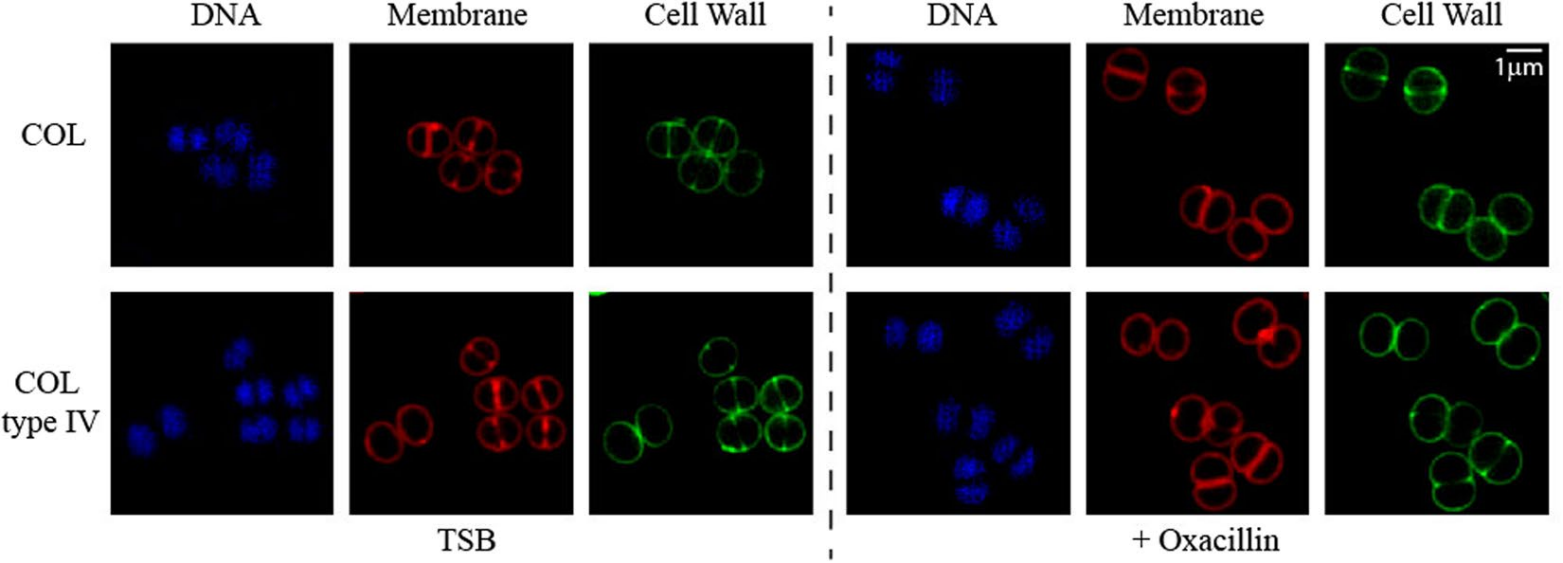
Structured Illumination Microscopy (SIM) of COL and COL type IV in response to oxacillin challenge. COL and COL type IV were grown to early exponential phase before incubation for 1 h with oxacillin (256 µg/ml and 512 µg/ml, respectively). Cell were next incubated with Hoechst3332 (blue), Nile Red (red) and a fluorescent derivative of vancomycin (green) in order to stain the DNA, cell membrane and cell wall, respectively. Visualisation by SIM shows that the cell morphology of COL type IV is similar to COL, both in the presence and absence of oxacillin.

The question we aimed to answer was whether the requirement of PBP4 for β-lactam resistance observed in type IV SCC*mec* CA-MRSA strains was encoded in the SCC*mec* cassette. In other words, if the exchange of SCC*mec* in COL affected resistance to oxacillin in the presence of cefoxitin. To this end, the MICs of cefoxitin for MW2, COL, and COL type IV were first evaluated by a population analysis profile (PAP) method, and determined to be 32 µg/ml, 256 µg/ml and 256 µg/ml, respectively. The synergistic activity of oxacillin and cefoxitin was then determined by PAP using increasing concentrations of oxacillin in the presence and absence of ¼ X MIC of cefoxitin (Fig. 3). COL type IV resistance to oxacillin was marginally affected by the addition of cefoxitin (twofold drop), mimicking COL, while the oxacillin MIC for MW2 dropped 32-fold in the presence of cefoxitin. These results were reinforced by genetically inactivating the key target of cefoxitin, PBP4, encoded by *pbpD*, in strains COL, COL type IV and MW2, and only observing an appreciable drop in oxacillin resistance in the MW2 background (Fig. 3). The expression levels of PBP2A were found to be maintained in all strains irrespective of PBP4 presence (Fig. 2). We also questioned whether there was any difference in the morphological response of COL and COL type IV to antibiotic challenge. To this end, COL and COL type IV were grown to early exponential phase before exposure to oxacillin for 1 hr, and visualised by SIM, having been incubated with DNA, membrane and cell wall staining dyes. Under these conditions, no differences in the morphological response to oxacillin were observed (Fig. 4).

Together, these data demonstrate that in the background of COL, type IV SCC*mec* does not confer changes in resistance against oxacillin in the presence of cefoxitin compared to type I SCC*mec*, and in turn, suggests that genetic elements pertinent to conferring resistance to the two β-lactams’ synergy reside outside of the SCC*mec* region.

Having the two types of SCC*mec* in the same background also gave us a unique opportunity to evaluate resistance to other antibiotics. Therefore, the MICs of cell wall, cell membrane, protein and DNA synthesis targetting antibiotics were also determined. As shown in Supplementary Table S3, the exchange of SCC*mec* in the COL strains had a minimal effect on resistance levels, occasionally conferring twofold differences in MIC. These results indicate that the genetic differences between SCC*mec* type I and IV do not play a role in resistance to any of the tested antibiotics.

### Exchange of SCC*mec* types in MW2

To support the data above, we wanted to perform the reverse SCC*mec* exchange and replace the SCC*mec* type IV of MW2 by the SCC*mec* type I of COL. For that purpose, we first had to construct a MW2 strain lacking its SCC*mec*. MW2 has previously been described as an excision deficient strain, since the overexpression of CcrAB2 proteins from SCC*mec* type IV strains was ineffective in MW2 SCC*mec* excision^35^. However, introduction of plasmid pSR encoding *ccrAB2* from N315 (SCC*mec* type II) was sufficient to induce the excision of the SCC*mec* type IV from MW2^10, 28^, giving rise to MW2-S. This strain was confirmed by sequencing and had an oxacillin MIC of 1 µg/ml (Fig. 1). There is the possibility that the introduction of SCC*mec* type I into MW2-S could eventually confer an increase in β-lactam resistance compared to the wild type strain, and so, as a safety precaution, we decided to modify this strain to reduce its virulence. To this end, we deleted the *spa* gene encoding the virulence factor Protein A, which has been shown to play important roles during infection in animal models^36, 37^. This strain, designated MW2-SΔ*spa*, was used as the recipient strain and COL and COL pSR as the donor strains for transductions with bacteriophage 80α. However, we were not able to obtain MW2 with SCC*mec* type I. Since the neighbouring regions of the *attB* site have been implicated in affecting the rates of integration and excision of the SCC*mec* cassette^35, 38^, we decided to introduce part (650 bp) of the immediate downstream region of the COL *attB* site, into MW2-SΔ*spa*, leading to strain MW2-SΔ*spa*0057 (Fig. 1). We hoped that this modification would increase the probability of SCC*mec* type I homologous recombination, but we were again unable to obtain recipient colonies with increased oxacillin resistance. Lastly, electrocompetent MW2-SΔ*spa*0057 cells were produced, as previously described^21^, and competence was confirmed by the introduction of a control plasmid pMAD. However, electroporation using chromosomal DNA of COL did not produce colonies following selection with 4 µg/ml oxacillin. We were therefore unable to introduce type I SCC*mec* into the MW2 background, despite numerous attempts.

## Discussion

The rise in the incidence of drug resistant strains of *S. aureus* over the past few decades, has led to a renewed effort not only to identify novel antibiotics, but also novel strategies to combat infections. The use of combination therapies has posed promising treatment alternatives, whereby the individual effectiveness of two already existing drugs is enhanced by the presence of the other. Since β-lactams are very effective antibiotics, the majority of combination therapy studies focus on the identification of drugs and cellular targets that will restore β-lactam sensitivity^39^. The most common combination therapy involves the targetting of the resistance adaptation directly, namely through the use of β-lactamase inhibitors. β-lactamases increase β-lactam resistance by binding to and hydrolysing the drug itself, and the use of β-lactamase inhibitors effectively reduces a strains’ MIC^40^. However, this strategy does not overcome PBP2A-dependent resistance. Alternative approaches have been proposed, including the disruption of the synthesis of other cellular components such as the glycopolymers lipoteichoic acid (LTA) and wall teichoic acid (WTA), which play pivotal roles in stabilising the cell surface of *S. aureus*. Examples include krisynomycin, an inhibitor of the signal peptidase SpsB that is required for the correct processing of the lipoteichoic acid synthase, LtaS, and targocil, an inhibitor of the WTA synthesis protein, TarG, involved in the translocation of the glycopolymer across the membrane^41–43^. Alternatively, the combined use of β-lactams and antibiotics that inhibit the earlier steps of peptidoglycan synthesis are highly effective, since together they require the bacterium to develop two distinct resistance mechanisms simultaneously, in order to survive. Fosfomycin, targetting the first enzyme responsible for the production of the peptidoglycan subunit, uridine diphosphate-N-acetylmuramic acid, and DMPI, thought to prevent the flipping of lipid II across the membrane, are just two such examples^44–46^. Although the frequency of resistance has been shown to be reduced in the above examples, the ability of *S. aureus* to acquire new resistance mechanisms is impressive, and so it is important to fully understand the mechanism of synergistic activity.

The study of antibiotic combinations at the origin of this work demonstrated synergistic activity between the PBP4-selective cefoxitin and other β-lactams, namely oxacillin^19, 20^. Given that both of these antibiotics work by preventing transpeptidation through the binding of PBPs, this result highlights the differential roles of the PBPs in peptidoglycan synthesis. What was surprising was that the synergy of oxacillin and cefoxitin was evident against CA-MRSA, but it did not extend to HA-MRSA strains^19^. Initial data pointed towards the SCC*mec* type as the cause of this difference, since type IV carrying strains correlated with susceptibility to oxacillin/cefoxitin synergy, while COL, a type I SCC*mec* containing HA-MRSA strain, maintained resistance. We therefore wanted to exchange the SCC*mec* type I and type IV, in order to analyse the resistance levels to oxacillin and cefoxitin in the same genetic background, thereby ascertaining whether the SCC*mec* type was in fact the key determinant.

Of the 12 types of SCC*mec* identified so far, type IV appears to have been acquired most frequently, existing in at least three independent *S. aureus* backgrounds^47, 48^. The relatively small size (24 kb) of the SCC*mec* type IV may mitigate its uptake, compared to the larger SCC*mec* types such as type I (34 kb)^48^. In addition, the carriage of type I SCC*mec* in *S. aureus* has been shown to generate a fitness cost, implying that without environmental pressure, the loss of this genetic element may be desirable^49^.

In this work, we began by introducing the SCC*mec* type IV of MW2 into the preexisting COL-S strain lacking the native SCC*mec* type I. This was performed by transduction using bacteriophage 80α, whose capacity to take up large genetic fragments had previously been shown^16^. Based on NGS and SNP analysis, not only was the 24 kb SCC*mec* transferred to COL-S, but most likely an additional 5 kb both upstream and downstream of the chromosomal integration site in MW2 was moved, allowing for homologous recombination. Transduction of the SCC*mec* element between *S. aureus* strain has already been published by Scharn *et al*.^50^, where both SCC*mec* types I and IV were successfully transduced to recipients, leading to an interruption of the OrfX gene. These authors set out to establish the bacterial requirements for transduction, highlighting the need to have the same arginine catabolic metabolic element (ACME) type and the presence of a penicillinase plasmid^50^. However, contrary to their data^50^, COL type IV was attainable in the absence of a penicillinase-producing plasmid. The construction of COL type IV not only renews the idea of transduction as a possible method for SCC*mec* transfer between *S. aureus* strains, but also provides an alternative mechanism to cassette chromosome recombinases for its incorporation into the genome. SCC*mec* elements are known to generate circular DNA elements following excision from the chromosomal DNA, ultimately ensuring that all genes necessary for its re-integration are present during transfer^2^. Our work reinforces the idea that homologous recombination of large mobile genetic elements including the SCC*mec* may occur in nature, potentially bypassing the need for the cassette chromosome recombinases. However, transduction of DNA between staphylococcal species has not been observed, and conjugation remains a likely mechanism for interspecies DNA distribution. Ray and colleagues demonstrated not only the capture of a truncated SCC*mec* element on a conjugative plasmid, but also the subsequent introduction into MSSA strains, reaffirming conjugation as a plausible theory for SCC*mec* acquisition^18^.

Once DNA uptake has occurred, site-directed recombinases mediate the integration and excision of the SCC*mec* element at the 3′ end of the *orfX* gene, the rate of which is affected by the *S. aureus* background. For instance, MW2 along with other SCC*mec* type IV carrying strains were classified as being excision deficient, since introduction of the MW2 specific cassette chromosome recombinases (CcrAB2) on a plasmid could successfully excise the SCC*mec* from COL (type I) and N315 (type II), but surprisingly not from MW2 itself^35^. In this work we successfully induced the loss of the SCC*mec* from MW2 via the expression of N315 CcrAB2. However, despite implementing several different strategies, all attempts to introduce the SCC*mec* type I of COL were unsuccessful. While the difficulty in SCC*mec* acquisition by these strains most likely occured during the uptake of the large 34 kb region, problems during the subsequent integration process cannot be ruled out. This was observed recently in the background of the MSSA strain RN4220, where the SCC*mec* element was transferred by conjugation, but instead of being integrated, it was maintained in the cell as circular DNA^18^.

The data presented in this paper demonstrates that in the background of COL, the exchange of SCC*mec* type I for SCC*mec* type IV did not lead to susceptibility to the oxacillin in the absence of PBP4, as observed in MW2. Concordantly, the addition of cefoxitin, a β-lactam with high affinity to PBP4, led to a two-fold drop in oxacillin resistance in COL and COL type IV, in stark contrast to the 32-fold reduction in oxacillin resistance observed in MW2. Furthermore, no significant differences in resistance levels to either β-lactam and non-β-lactam antibiotics were identified between COL and COL type IV. Therefore we conclude that despite the previously observed correlation, the SCC*mec* element is unlikely to be the principal determinant in resistance to the synergistic activity of oxacillin and cefoxitin. Instead, this work suggests that factors residing outside of the SCC*mec* element of COL are key in understanding its ability to resist β-lactam synergy and their identification may facilitate the establishment of alternative drug combinations or even novel antibiotic targets.

## Electronic supplementary material

Suplementary data
