# Supplementary material for: Role of SCCmec type in resistance to the synergistic activity of oxacillin and cefoxitin in MRSA
Source: Sci Rep. 2017 Jul 21;7:6154. doi: 10.1038/s41598-017-06329-2 (PMC5522475; doi:10.1038/s41598-017-06329-2)
Supplement: Supplementary file 1 — Suplementary data [file 41598_2017_6329_MOESM1_ESM.doc]

**Role of SCC*mec* type in resistance to the synergistic activity of oxacillin and cefoxitin in MRSA**

Nathalie T. Reichmann^1^ and Mariana G. Pinho^1^*

^1^Instituto de Tecnologia Química e Biológica António Xavier, Universidade Nova de Lisboa, Portugal

Key words: *Staphylococcus aureus*, SCC*mec*, penicillin-binding proteins, β-lactams, antibiotic synergy

Running title: Role of SCC*mec* type in resistance to β-lactam synergy

* Corresponding author. Mailing Address: Laboratory of Bacterial Cell Biology,

Instituto de Tecnologia Química e Biológica António Xavier, Universidade Nova de Lisboa, Avenida da República, Apartado 127, 2781-901 Oeiras, Portugal. Email: mgpinho@itqb.unl.pt

**SUPPLEMENTAL DATA**

TABLE S1: Primers used in this study.

| **Number** | **Primer** | **Sequence** |
| --- | --- | --- |
| 1 | EcoRI-UpSpa(600bp) For | cgcgaattcctggttcagttgtaaataac |
| 2 | UpSpa-DownSpa Rev | gggggtattaataaacaaacaatacacaacgatag |
| 3 | UpSpa-DownSpa For | gtattgtttgtttattaataccccctgtatgtatttg |
| 4 | DownSpa(600bp)-BamHI Rev | cgcggatccgttagagctctcaataatttaa |
| 5 | BglII-UpOrfX For | cgagatctgaaatacccacaatgcccacag |
| 6 | OrfX-UpSACOL0057 Rev | gttttacttatgatacgcctctccacgcataatc |
| 7 | OrfX-UpSACOL0057 For | gtggagaggcgtatcataagtaaaactaaaaaattc |
| 8 | DownSACOL0057-MW2attB Rev | gaacctcattacattacaccccttaattatattttac |
| 9 | DownSACOL0057-MW2attB For | aaggggtgtaatgtaatgaggttcatgatttttgac |
| 10 | MW20048 (440bp) Rev | cgcggatccgcgtaataaaatcttccag |
| 11 | pSR1 SeqI | gttggagctcaggtcgattc |
| 12 | pSR1 SeqII | gcaacgcactacatctatcaatg |
| 13 | OrfX 5 end For | gaaaatcaccattttagc |

Restriction sites are underlined**.**

**TABLE S2: SNPs identified in COL type IV compared to COL.**

| **Genomic Location** | **Description** | **DNA Mutation** | **AA mutation** |
| --- | --- | --- | --- |
| 31171 | *sasH* | A-G | K49R |
| 31210 | *sasH* | C-A | S62Y |
| 31214 | *sasH* | CA-TG | K64E |
| 31273 | *sasH* | G-A | S83N |
| 31296 | *sasH* | T-A | S91T |
| 31308 | *sasH* | T-A | S95T |
| 31315 | *sasH* | T-C | V97A |
| 31327 | *sasH* | A-C | K101T |
| 31330 | *sasH* | T-G | V102G |
| 31335 | *sasH* | G-A | E104K |
| 31339 | *sasH* | C-G | T105R |
| 31342 | *sasH* | G-A | R106H |
| 31346 | *sasH* | C-T |  |
| 31353 | *sasH* | A-G | T110A |
| 31394 | *sasH* | G-A |  |
| 31453 | *sasH* | C-T | A143V |
| 31625 | *sasH* | C-T |  |
| 31628 | *sasH* | G-A |  |
| 31817 | *sasH* | A-G |  |
| 31847 | *sasH* | T-C |  |
| 31898 | *sasH* | C-T |  |
| 31958 | *sasH* | C-T |  |
| 31982 | *sasH* | A-T |  |
| 31991 | *sasH* | A-T |  |
| 32024 | *sasH* | T-C |  |
| 32081 | *sasH* | T-G |  |
| 32087 | *sasH* | T-C |  |
| 32102 | *sasH* | A-C |  |
| 32141 | *sasH* | G-A |  |
| 32143 | *sasH* | C-T | A373V |
| 32204 | *sasH* | G-A |  |
| 32219 | *sasH* | A-G |  |
| 32318 | *sasH* | T-A |  |
| 32327 | *sasH* | A-T |  |
| 32339 | *sasH* | C-T |  |
| 32345 | *sasH* | C-G |  |
| 32426 | *sasH* | G-A |  |
| 32615 | *sasH* | C-T |  |
| 32633 | *sasH* | G-A |  |
| 32670 | *sasH* | C-T |  |
| 32690 | *sasH* | C-T |  |
| 32708 | *sasH* | A-G | I561M |
| 32776 | *sasH* | A-G | N584S |
| 32801 | *sasH* | C-T |  |
| 32834 | *sasH* | C-A |  |
| 32858 | *sasH* | T-C |  |
| 32935 | *sasH* | C-T | T637M |
| 32971 | *sasH* | C-T | A649V |
| 32974 | *sasH* | T-C | V650A |
| 33018 | *sasH* | A-G | K665E |
| 33048 | *sasH* | G-A | D675N |
| 33050 | *sasH* | C-A | D675E |
| 33053 | *sasH* | C-G | D676E |
| 33063 | *sasH* | G-A | D680N |
| 33075 | *sasH* | A-C | K684Q |
| 33083 | *sasH* | TC-GA | P687T |
| 33098 | *sasH* | A-G |  |
| 33104 | *sasH* | G-A |  |
| 33106 | *sasH* | T-C | L694P |
| 33148 | *sasH* | G-A | G708D |
| 33153 | *sasH* | A-G | T710A |
| 33156 | *sasH* | A-T | I711L |
| 33165 | *sasH* | G-A | A714T |
| 33168 | *sasH* | A-G | T715A |
| 33179 | *sasH* | C-T |  |
| 33202 | *sasH* | GA-AC | R726N |
| 33211 | *sasH* | T-C | V729A |
| 33227 | *sasH* | G-A |  |
| 33258 | *sasH* | A-G | N745D |
| 33277 | *sasH* | A-C | E751A |
| 33294 | *sasH* | T-G | L757V |
| 33297 | *sasH* | G-A | A758T |
| 33358 | *sasH* downstream | T-C |  |
| 33407 | *sasH* downstream | A-G |  |
| 33416 | *sasH* downstream | G-T |  |
| 33449 | *sasH* downstream | A-G |  |
| 33474 | *sasH* downstream | G-A |  |
| 33553 | *sasH* downstream | C-T |  |
| 33567 | *sasH* downstream | G-A |  |
| 33578 | *sacol0025* | T-C | .160Q |
| 33584 | *sacol0025* | G-A | D158N |
| 33591 | *sacol0025* | T-C |  |
| 33600 | *sacol0025* | C-T |  |
| 33624 | *sacol0025* | AT-TG | I144S |
| 33645 | *sacol0025* | G-T |  |
| 33826 | *sacol0025* | G-T | G77V |
| 33855 | *sacol0025* | A-G |  |
| 33867 | *sacol0025* | A-G |  |
| 34023 | *sacol0025* | A-T | K11N |
| 34071 | *sacol0025* upstream | C-T |  |
| 34107 | *sacol0025* upstream | C-T |  |
| 34170 | *sacol0025* upstream | A-T |  |
| 70970 | *sacol0060* | CC-TT | R29. |
| 70983 | *sacol0060* | C-T |  |
| 71169 | *sacol0061* | G-A | V247I |
| 72257 | *sacol0062* upstream | G-A |  |

|  | **COL** | **COL type IV** | **MW2** |
| --- | --- | --- | --- |
| **Cephradine** | 256 | 512 | 256 |
| **Vancomycin** | 4 | 4 | 2 |
| **Bacitracin** | 256 | 256 | 256 |
| **D-cycloserine** | 256 | 256 | 128 |
| **Daptomycin** | 2 | 2 | 1 |
| **Chloramphenicol** | 8 | 8 | 8 |
| **Nalidixic acid** | 512 | 512 | 64 |

**TABLE S3. Antibiotic Minimum Inhibitory Concentrations assessed by microdilution.** Overnight cultures of COL, COL type IV, and MW2 were diluted to a final OD_600nm_ of 0.0025 and grown in the presence of twofold dilutions of various antibiotics at 37°C for 48 h. The lowest concentration of antibiotic at which growth was prevented is defined as the Minimal Inhibitory Concentration (MIC). Experiments were performed a minimum of three times and the mode value is presented here.
